# Teeport: Break the Wall Between the Optimization Algorithms and Problems

**DOI:** 10.3389/fdata.2021.734650

**Published:** 2021-11-16

**Authors:** Zhe Zhang, Xiaobiao Huang, Minghao Song

**Affiliations:** ^1^ SLAC National Accelerator Laboratory, AD SPEAR3 PCT Accel Physics, Menlo Park, CA, United States; ^2^ Department of Physics, Illinois Institute of Technology, Chicago, IL, United States

**Keywords:** optimization, real-time communication, benchmarking, remote, platform

## Abstract

Optimization algorithms/techniques such as genetic algorithm, particle swarm optimization, and Gaussian process have been widely used in the accelerator field to tackle complex design/online optimization problems. However, connecting the algorithm with the optimization problem can be difficult, as the algorithms and the problems may be implemented in different languages, or they may require specific resources. We introduce an optimization platform named Teeport that is developed to address the above issues. This real-time communication-based platform is designed to minimize the effort of integrating the algorithms and problems. Once integrated, the users are granted a rich feature set, such as monitoring, controlling, and benchmarking. Some real-life applications of the platform are also discussed.

## 1 Introduction

As accelerators push the performance limit, for example, in terms of beam emittance or brightness, the controls become more complex. Realizing the design performance in spite of the many inevitable imperfections in the real machines is very challenging. In recent years, it has become a trend for accelerator physicists to resort to online optimization, i.e., directly optimizing the control parameters of the machines with computer algorithms during operation, to bring out the best machine performance and reduce tuning time (Huang et al., 2013; Pang and Rybarcyk, 2014; Tian et al., 2014; Huang, 2016; Olsson, 2018; Bergan et al., 2019; Duris et al., 2020).

In a typical online optimization scenario, the evaluation script that controls the machine parameters and reads or calculates the objective to be optimized usually lives in the accelerator control room (ACR), while the codes of optimization algorithms are copied to the same computer in the ACR and adapted to the evaluation script and perform the optimization task there. There are a few problems posed by this simple and straightforward method. If the optimization algorithm was tested in a simulation setup and then copied to the ACR, some re-configuration may be needed, such as adapting the API to the experimental evaluation script and setting up the algorithm run-time environment. These seemingly trivial tasks may be complicated, time-consuming, and error-prone. This work may need to be done each time a new algorithm is used or a new experimental problem is optimized. Furthermore, it could be a daunting task to connect the algorithm and the evaluation scripts if they are written in different languages. Sometimes for security considerations, an externally developed algorithm run-time environment is not allowed to be deployed in the ACR.

In this study, we developed an online optimization platform, Teeport, to addresses the aforementioned communication difficulties between the optimization algorithms and application problems. It is task-based, extensible, embeddable, and can be used for optimization and real-time testing. With Teeport, the algorithms and problems can be effortlessly integrated into a real-time messaging service, which gives the ability for the two sides to talk to each other freely. In addition, once integrated, the users are automatically granted a rich optimization-related feature set, including optimization process controlling, monitoring, comparing, and benchmarking. Teeport has been applied to solve real-life remote optimization tasks in several national laboratories in the US, including SLAC National Accelerator Laboratory (SLAC) and Argonne National Laboratory (ANL).

This paper is organized as follows. Section 2 introduces important concepts and philosophy in Teeport; Section 3 describes the key designs of Teeport, as well as discusses some implementation details to illustrate the way Teeport works; Section 4 highlights a few features that distinguish Teeport from the other optimization platforms; Section 5 shows several applications of Teeport to demonstrate what Teeport could do and how to apply Teeport to solve real-life optimization related problems; Section 6 concludes the paper and points out the future work on Teeport.

## 2 Philosophy

Several commonly used terms like optimization algorithms, optimization problems, and optimization process, could look and work very differently in different situations. The way to apply an algorithm to optimize a problem in application could also differ as a function of time which brings confusion and frustration. To make the various algorithms and problems work together consistently, Teeport introduces a few concepts to abstract the optimization-related objects. We’ll analyze the following real-life online optimization case and discuss the important concepts in Teeport along the way.

### 2.1 Evaluator

Assume that we have a Matlab script that reads and writes the process variables (PVs) through EPICS (Dalesio et al., 1991). When the optimization algorithm evaluates a solution (a point in the parameter space), the script writes the PVs with the values given by the algorithm, then reads and returns the PV value of the objective. There could be some configurable parameters during the evaluation, such as the waiting time between the PV writing and reading. Therefore, the whole evaluation process can be abstracted as a function:

$$Y=evaluate\left(X,con\mathrm{fi}gs\right)$$ (1)

Here **X** and **Y** are 2D arrays, have a shape of (*n*, *v*) and (*n*, *o*) respectively, where *n* denotes the number of the points to be evaluated, *v* the number of the variables, and *o* the number of the objectives. The *n* points that are passed into the evaluate function through the **X** array are called a generation. The concept of generation in Teeport is different from its usual definition in evolutionary algorithms–here one generation means a batch of data points that could be evaluated simultaneously, there is no order requirement when evaluating them. A generation in Teeport could contain only one data point^1^, while a generation with only one individual generally does not make sense in an evolutionary algorithm like NSGA-II. Any evaluation process, including simulations running on a laptop, parallel evaluation tasks running on a cluster, and experiments on a real machine, could be abstracted as the evaluate function as shown in Eq. 1. In Teeport, we call the evaluation process that has been implemented in the form shown in Eq. 1 an evaluator.

### 2.2 Optimizer

On the other hand, assume the optimization algorithm is a Python script that imports several optimization-related packages, that accept an evaluate function and tries to optimize it. The algorithm usually takes in parameters such as the dimension of the problem to be optimized, the number of the objectives, and parameters related to the termination conditions. The optimization algorithm can be abstracted like this:

$$\left[X_{\text{opt}},Y_{\text{opt}},\ldots ,\right]=optimize\left(evaluate,con\mathrm{fi}gs\right)$$ (2)

Where [**X**_opt_, **Y**_opt_, …, ] are optional return arguments. Any optimization process, including multi-objective genetic algorithms (MOGA), Gaussian process (GP) optimizer, and even a human operator who decides which data points to be evaluated in the next step, could be abstracted as such an optimize function as shown in Eq. 2. In Teeport, we call the optimization process implemented in the form shown in Eq. 2 an optimizer.

### 2.3 Adapter

**FIGURE 1:**
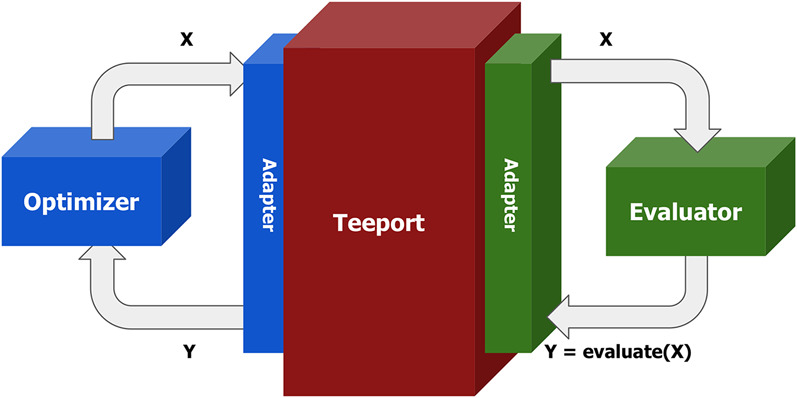
Schematic plot of an optimization loop in Teeport. With the help of the adapters provided by Teeport, the optimizer and the evaluator can exchange data in a normalized format.

As discussed above, the evaluator and the optimizer may be implemented in different languages. To enable them to talk to each other, Teeport provides an adapter, or client, for each language, and a messaging engine as a middleware between the evaluator and the optimizer. With the corresponding adapter, the data flowing in and out of the optimizer and the evaluator will be normalized to 2D real number arrays^2^ encoded in a JSON string and subsequently forwarded by the messaging engine to complete the optimization loop. The process is as illustrated in Figure 1.

### 2.4 Monitor/Controller

**FIGURE 2:**
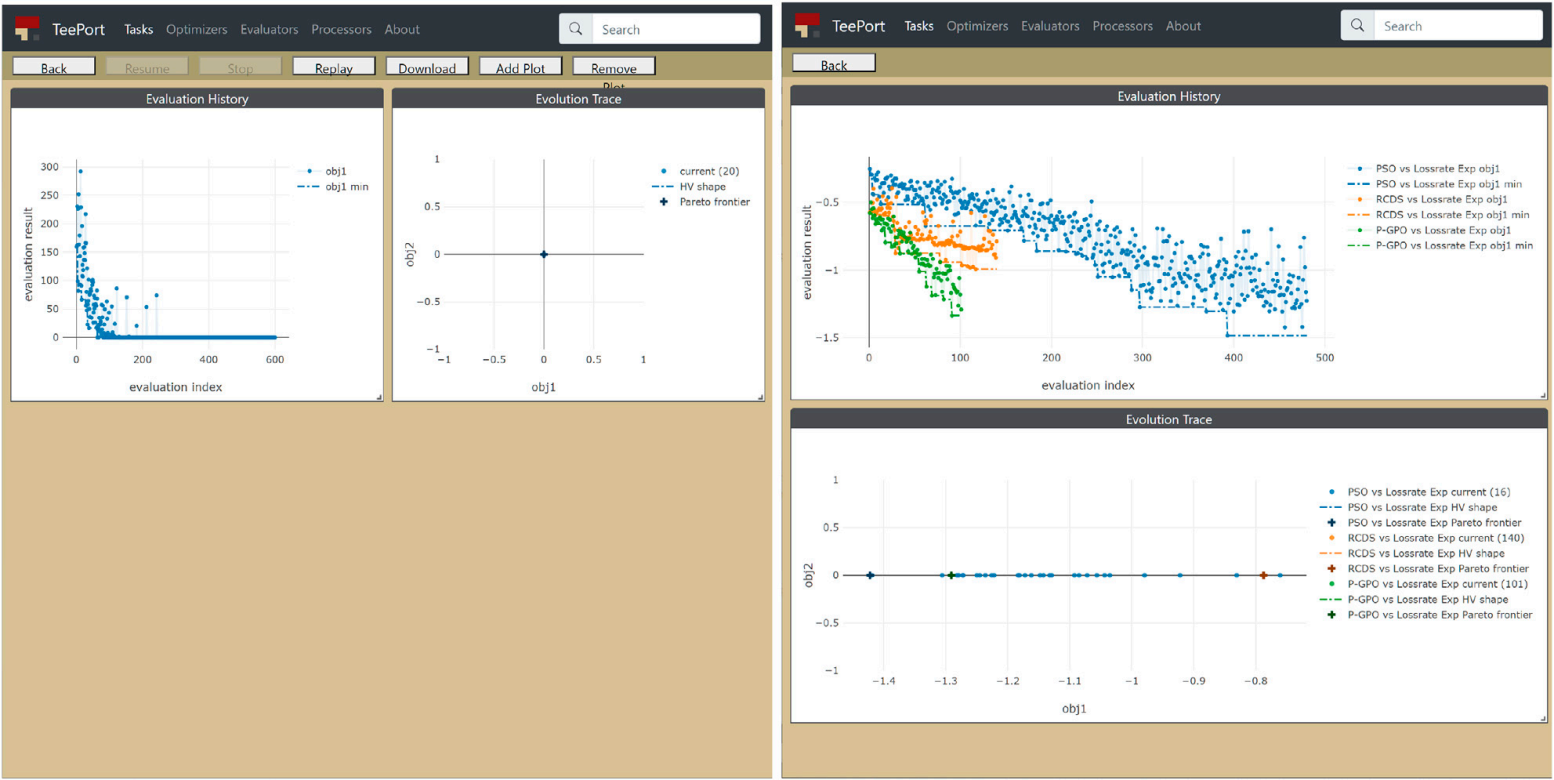
Left: the history data of an online optimization experiment that was performed through Teeport; Right: comparison among the performance of three optimization algorithms against the SPEAR3 beam loss rate online optimization problem.

The optimization data flow through the Teeport messaging middleware. One can add the control and monitor layers to the middleware, to make the online optimization more controllable and visible. A visualization of the optimization process based on the data flow is called a monitor and is provided by the Teeport GUI through a browser. Examples of monitors provided by Teeport are shown in Figure 2. Similarly, The set of functionalities that controls the optimization data flow is called a controller. The toolbar in Figure 2 that contains a row of buttons (“Resume,” “Stop,” etc) is the controller for that task.

### 2.5 Processor

There are usually two participants in an optimization loop: the evaluator and the optimizer, as we have discussed above. However, sometimes it is useful to have a helper function that does not participate directly in the optimization loop. Let’s imagine the following situation. We are developing a new optimization algorithm in Matlab, which involves a modeling step. The best modeling package is, unfortunately, available only in Python. It would be ideal if we could use the Python modeling package to complete the modeling step, and perform all the rest steps in Matlab. The function that is written in Python and does the modeling job, is called a processor in the Teeport framework.

Processor could be treated as an extension of the evaluator concept, the process function has a similar signature compared to the evaluate function, as shown below:

$$results=process\left(data,con\mathrm{fi}gs\right)$$ (3)

The only difference is that for the process function, the input argument data and the returned value results are not limited to 2d arrays, they could be any serializable data, such as a dictionary, a list, a JSON object, etc. With a processor that is implemented in the form of Eq. 3, the aforementioned issue could be easily resolved by integrating the processor into Teeport, and remotely calling it within the optimization algorithm. It’s worth noting though, one of the main differences between processors and evaluators is that the processors are transparent to the optimization tasks. The data flowing through a processor is not related to any optimization tasks, and thus would not be cached and/or stored by Teeport. A processor can be used directly without initializing an optimization task–while an evaluator is always running within an optimization task. This flexibility of a processor makes it easy to fit into either side of the optimization task: The process function can be called in an evaluator, or in an optimizer, or even in another processor. An optimization task could have multiple processors to help with the computations, but only one evaluator is allowed in the task.

### 2.6 Design Principles

With the concepts introduced in the previous sections, we can now describe the Teeport design principles. The core philosophy of Teeport is to completely decouple the optimization algorithm and the problem to be optimized. By doing so, the algorithm developer does not need to care about the details of the optimization configuration (say, how to set up the problem to be compatible with the algorithm, where to put the algorithm code, etc). Instead, one could focus on the optimization logic (the optimize function) that really matters. On the other hand, the algorithm used does not have to figure out the usage of the optimization algorithm, which could vary greatly across different algorithms. The only thing that needs to be done on the user’s side is to write an evaluate function in one’s preferred/available language. Once the evaluator and the optimizer are available, Teeport will handle the rest.

**FIGURE 3:**
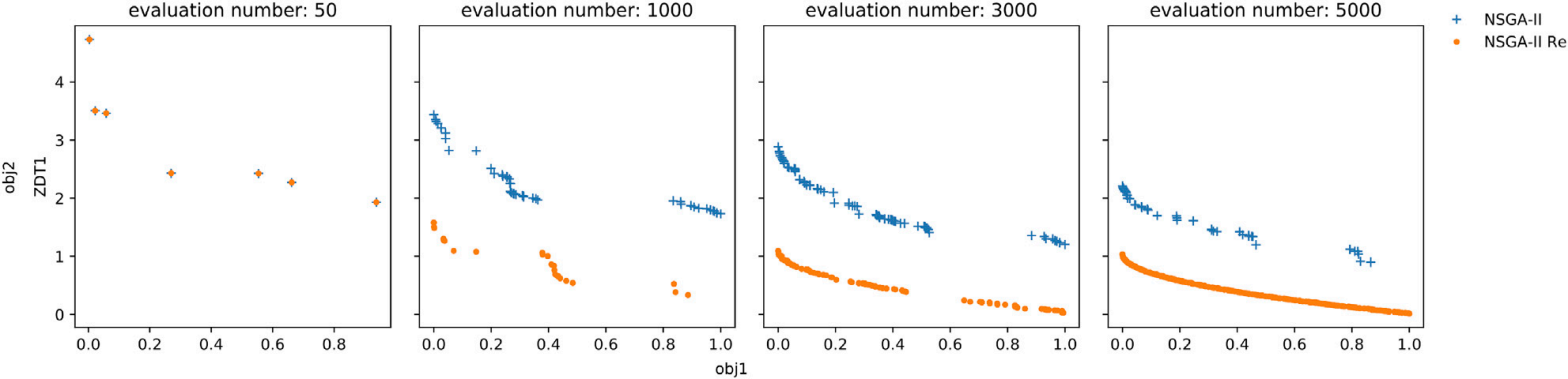
Performance comparison between the original NSGA-II in C (orange dot) and a popular NSGA-II implementation in Matlab (blue cross). Each plot shows the Pareto fronts of the two algorithms at a certain evaluation number (number of evaluated individuals in the optimization process). The initial population is identical for the two algorithms, as indicated in the leftmost plot. The two objectives are to be minimized so a lower Pareto front is preferable. It’s obvious that the original NSGA-II performs much better than the Matlab one along the whole optimization process.

Another principle that drove the design of Teeport is that the algorithm should be kept as original as possible. A popular approach to applying the same famous optimization algorithm on problems that are written in different languages is to port the algorithm to the target language. This porting process could bring in hard-to-detect errors that would cause significant performance issues under particular circumstances. Figure 3 demonstrates a real-life example. We compared the performance of the original NSGA-II (Deb et al., 2002) in C and a popular (downloaded more than 40,000 times since published) NSGA-II implementation in Matlab (Seshadri, 2009, since) against the ZDT1 (Zitzler et al., 2000) test problem. As shown in the plots, the Matlab version performed much worse than the original one. After a careful examination, the authors found the issue in the Matlab implementation–one line of code is missing that causes the incomplete gene mixing in the offspring. Therefore, it is not guaranteed that when you use a ported version of some specific optimization algorithm, the performance would be identical to the original one. Teeport resolves this problem by using the original algorithm/problem directly as it provides an adapter for each language so that all the algorithms/problems written in that language could be integrated effortlessly. It is much more efficient and practical compared to porting one algorithm to each language.

The third principle is to keep the interfaces minimal. It not only provides a minimal number of APIs but also minimizes the number of modifications that are needed to make the user’s code work with Teeport. In the simplest but still typical case, the user only needs to change 2 lines of code to integrate the optimization algorithm/problem into Teeport.

In the next section, we discuss the implementation details that enable Teeport to meet the above principles.

## 3 Key Designs

### 3.1 Task-Based Optimization

**FIGURE 4:**
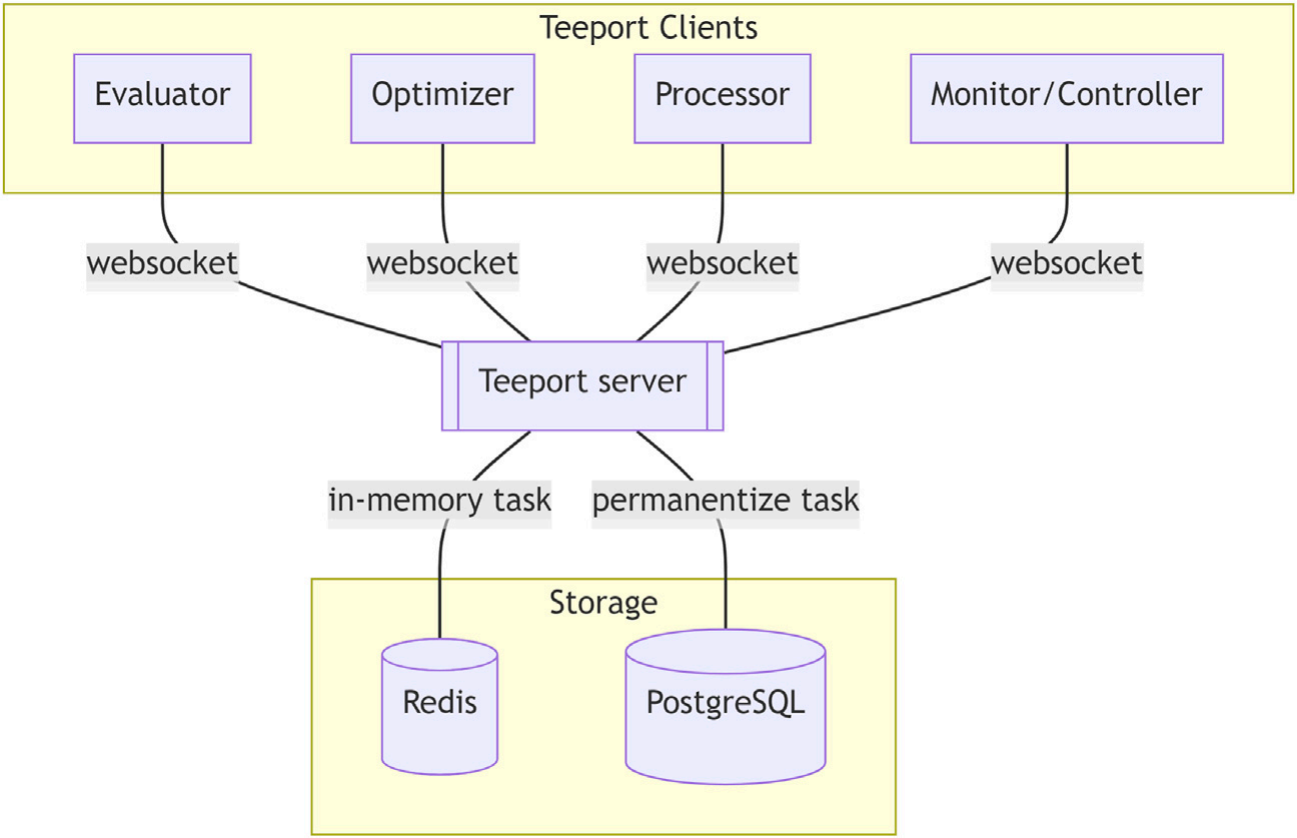
Architecture of Teeport. The Teeport clients are connected to the Teeport backend server through the WebSocket protocol. The Teeport backend server processes and forwards the messages between the clients, while storing the optimization related data that flows through it in the storage, such as the Redis and PostgreSQL database for data persistence.

Teeport connects the evaluators, the optimizers, the monitors, and the processors through a real-time communication (RTC) protocol so that they can exchange information with each other in real-time. The architecture of Teeport is illustrated in Figure 4. As shown in the architecture, the various clients connect to the Teeport backend service through the websocket (Fette and Melnikov, 2011) protocol. To group the clients by the optimization process so that the messages are forwarded to the expected targets, Teeport employs the task concept.

**FIGURE 5:**
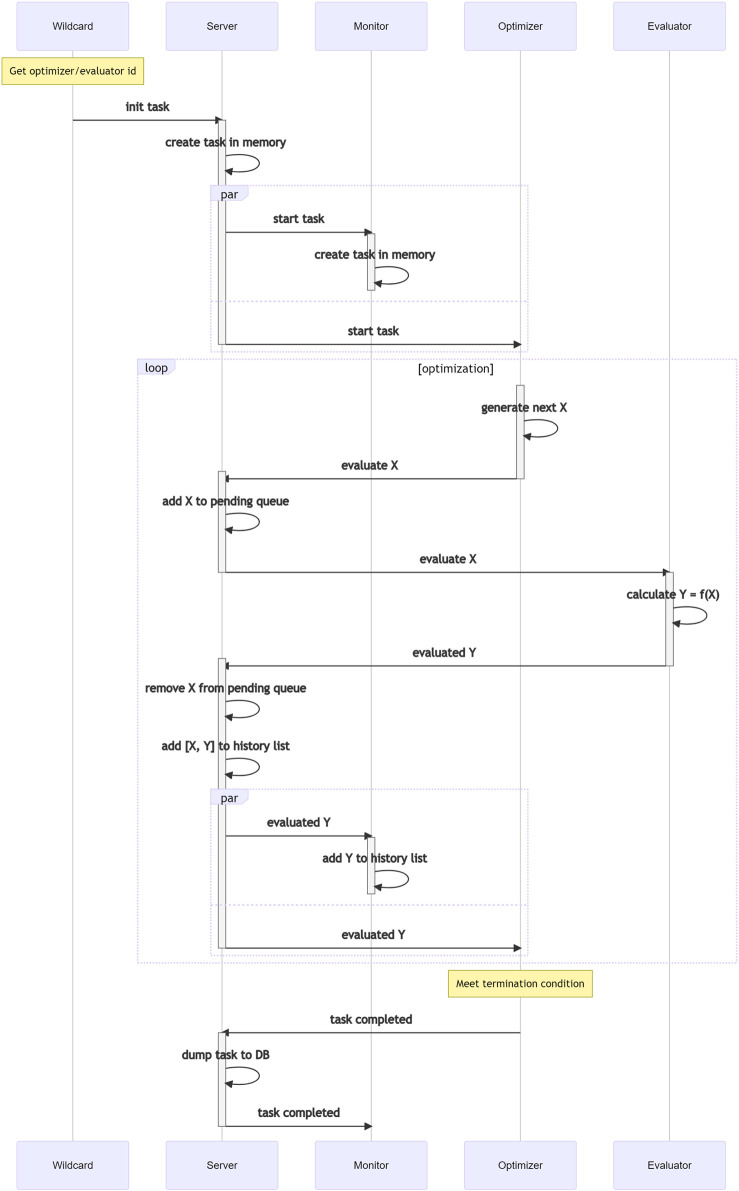
Task-based optimization data-flow in Teeport.

In Teeport, every optimization process is a task. A task needs at least two participants: the evaluator and the optimizer. Each task will be assigned a unique Id when initialized, and the messages between the evaluator and the optimizer will carry this Id information along the whole optimization process. The task-based optimization data flow is shown in Figure 5.

When an optimization task starts, the Teeport backend service will send a signal to the optimizer, the optimizer then calls its optimize function to start the optimization process. The optimize function would decide which data points (**X**) in the decision space to evaluate in the next step, and the data **X** is sent to the Teeport server, which is in turn forwarded to the corresponding evaluator. The evaluator calls its evaluate function to evaluate **X**, obtains the returned result **Y** and sends it to the Teeport server. The evaluated result **Y** is then forwarded back to the optimizer to complete one evaluation loop of the optimization. The optimization loop is repeated until the termination condition (which is usually coded in the optimize function) is satisfied.

Since the Teeport backend service forwards the messages between the clients to enable the optimization data flow, it could control the optimization process by holding the messages to be forwarded. It means that once the optimization algorithm and problem are integrated into Teeport, Teeport automatically grants the user the ability to pause, resume and stop the optimization process, without any additional code on the user’s side.

The optimization data **X** and **Y** that flows through the Teeport backend server are monitored by the monitors, to provide a real-time optimization process visualization to the users. The data are also temporarily stored in the Redis database and are archived in the PostgreSQL database after the optimization task is done, as shown in Figure 5.

### 3.2 Minimal Interfaces

Teeport defines a set of interfaces to deal with various situations.

For algorithm developers:

• Use an evaluator: evaluate_T: function = use_evaluator (id: string)
• Ship an optimizer: id: string = run_optimizer (optimize: function)
• Monitor an optimizer: optimize_T: function = use_optimizer (optimize: function)

For algorithm users:

• Use an optimizer: optimize_T: function = use_optimizer (id: string)
• Ship an evaluator: id: string = run_evaluator (evaluate: function)
• Monitor an evaluator: evaluate_T: function = use_evaluator (evaluate: function)

For both:

• Use a processor: process_T: function = use_processor (id: string)
• Ship a processor: id: string = run_processor (process: function)

The basic idea behind these APIs is to make it effortless to convert an evaluator/optimizer/processor from a local one to a remote one, and vice versa. Assume that you have your optimization algorithm (optimize function) locally on your laptop, and you would like to optimize a remote evaluator. The Teeport way to accomplish this is to use the use_evaluator (id) API to get a local version evaluate_T (a local function that is returned by the Teeport API may be called a Teeportized function) of that remote evaluate function. Since function evaluate_T is just a regular local evaluator that is written in the same language as your algorithm, the optimization task can be performed by calling optimize (evaluate_T) directly. A similar workflow applies to the situation that you have a local evaluator that waits to be optimized by a remote optimizer: just get the Teeportized optimizer and perform the optimization task locally as usual.

To ship or share a local evaluator/optimizer/processor is also straightforward: just use the run_evaluator (evaluate), run_optimizer (optimize), or run_processor (process) API accordingly to convert the local function to a remote one. These APIs will return an Id, which could be used to refer to the remote evaluator/optimizer/processor when someone wants to use your function.

**FIGURE 6:**
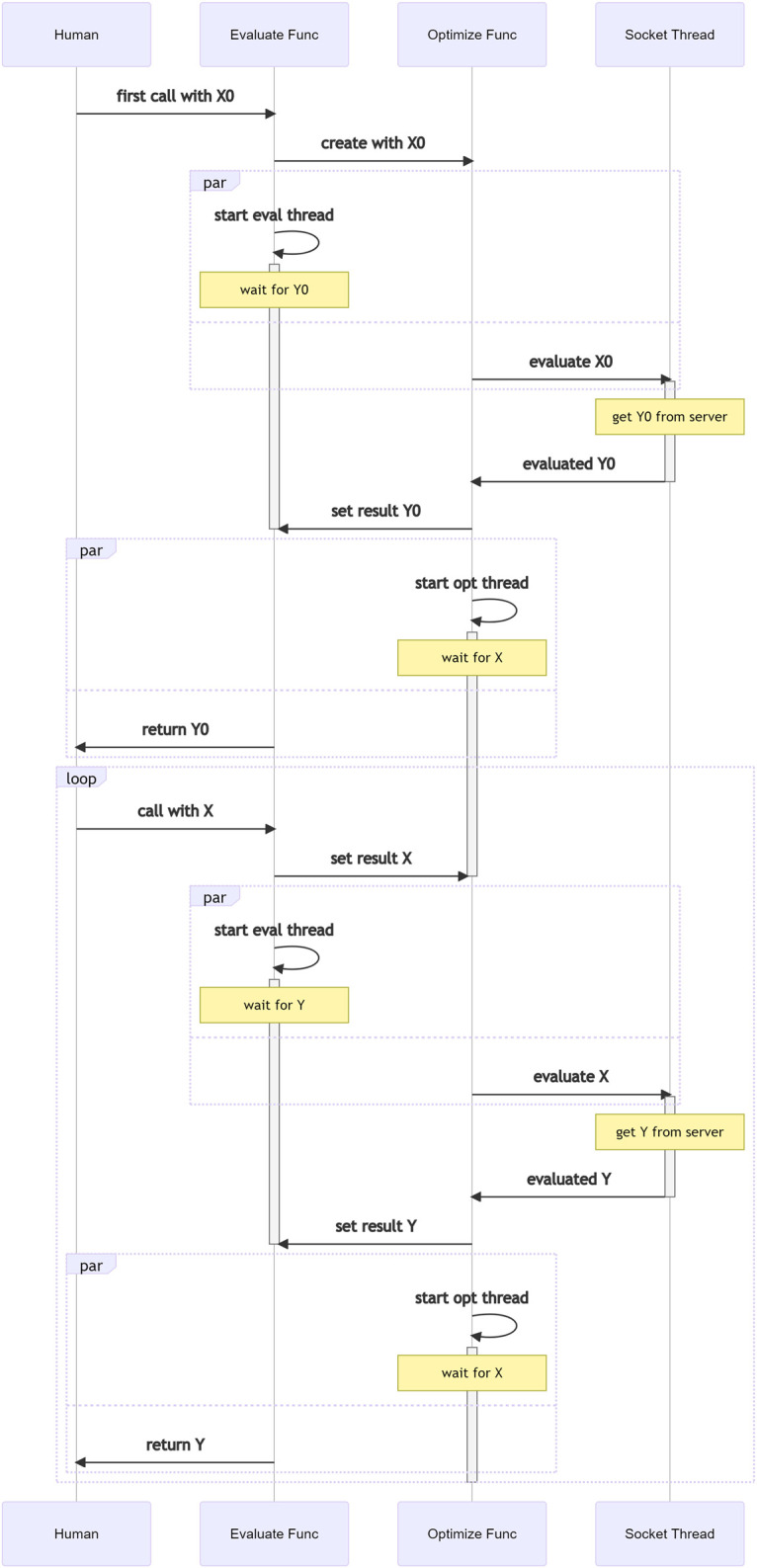
Multi-threading logic that happens when calling the Teeportized evaluate function from the use_evaluator (id) API to get some data **X** evaluated.

**FIGURE 7:**
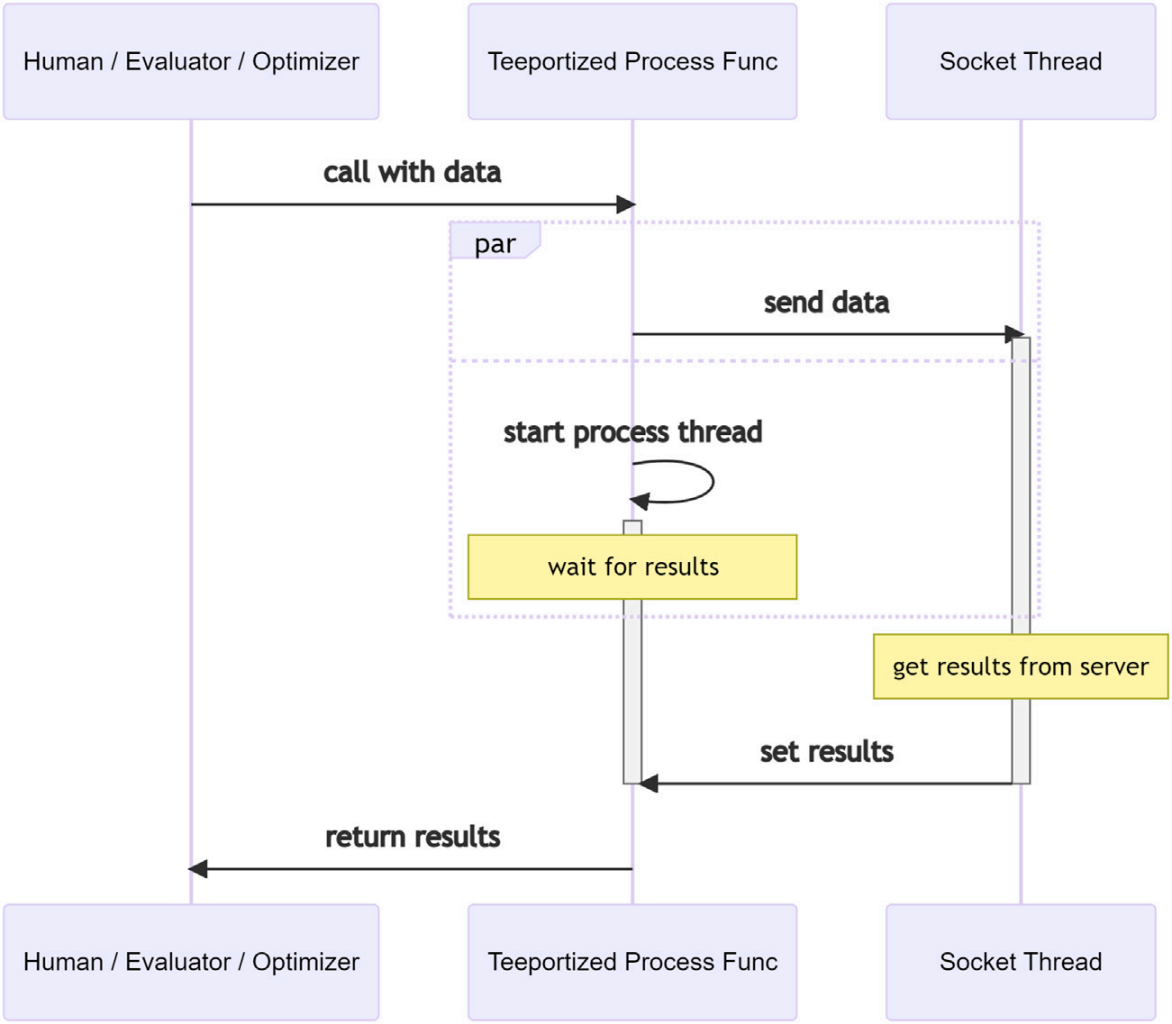
Data-flow in Teeport when calling the Teeportized process function from the use_processor (id) API to get some data processed.

It is worth noting that when using a Teeportized function in an optimization task, the actual calculation still happens where the remote function lives in, even though it just feels like it is the Teeportized function that does the calculation. This illusion is by design to minimize the impact of Teeport on the existing code/workflow. The internal logic in Teeport that creates this illusion when a Teeportized evaluator being called is shown in Figure 6. As mentioned in Section 2.5, a processor is not related to any optimization task. The data flow when a Teeportized process function gets called by a human being/evaluator/optimizer is shown in Figure 7, note the much simpler internal logic compared to Figure 6, which is the consequence of being independent of the optimization tasks.

**FIGURE 8:**
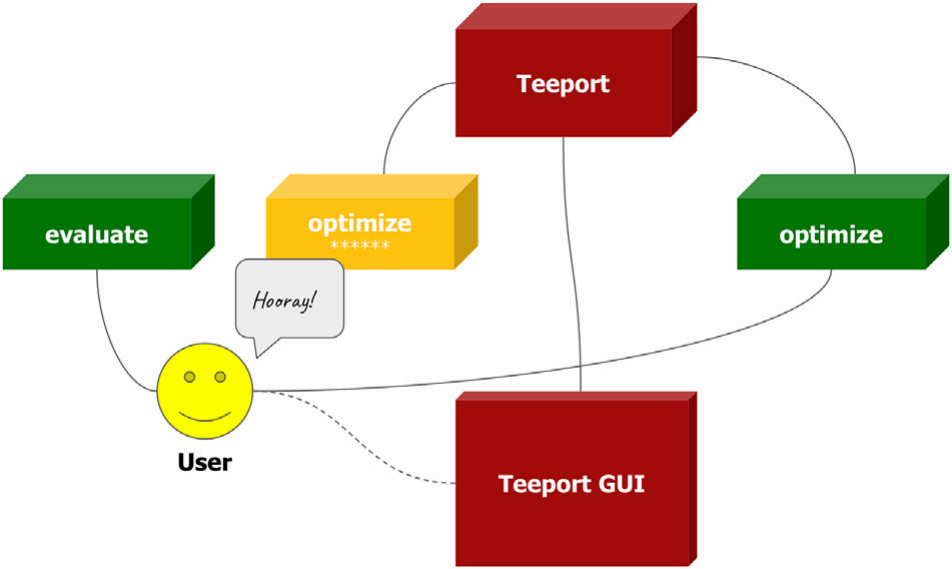
Using Teeport to monitor the optimization process without writing visualization-related code. The user in this cartoon used the use_optimizer (optimize) API upon the optimize function (green cube) to get a Teeportized optimize function (yellow cube). An alternative way to achieve the same goal is to wrap the evaluate function.

The monitoring usage of the Teeport APIs could look a little confusing, as the APIs accept a local function and return a Teeportized function. Let’s assume the following scenario where both the optimization algorithm and the problem are available locally and we would like to monitor the optimization process in real-time without writing any visualization code. In such a case the use_evaluator (evaluate) or use_optimizer (optimize) APIs are useful to wrap either the evaluate function or optimize function with the corresponding API to get a Teeportized version, and use it to perform the optimization normally. The optimization process will then be monitored in real-time on the Teeport GUI, as shown in Figure 8.

### 3.3 Fully Decoupled Frontend and Backend

All Teeport features except the optimization controller/monitor are available with the Teeport backend service. On top of that, Teeport ships a default web-based GUI to enhance the user experience under some common situations, such as benchmarking optimization algorithms, testing user-developed algorithms against different problems, or simply monitoring the optimization process. While the GUI is very useful, it is optional–the Teeport backend service is designed to be a standalone service that could work without a frontend.

The frontend and backend of Teeport are completely decoupled. The Teeport backend service provides a set of standard APIs to enable the clients to control and monitor the optimization process through WebSocket. Those APIs include:

• List all the optimizers/evaluators/processors (including the configurations)
• List all the tasks
• Get a detailed view of one specific task (including the history data)
• Create a new task
• Start/pause/resume/terminate a task
• Archive/restore a stopped task
• Update the metadata (name, description, etc) of a task
• Subscribe the updates of a task (including the metadata changing, the optimization data updating, etc)

**FIGURE 9:**
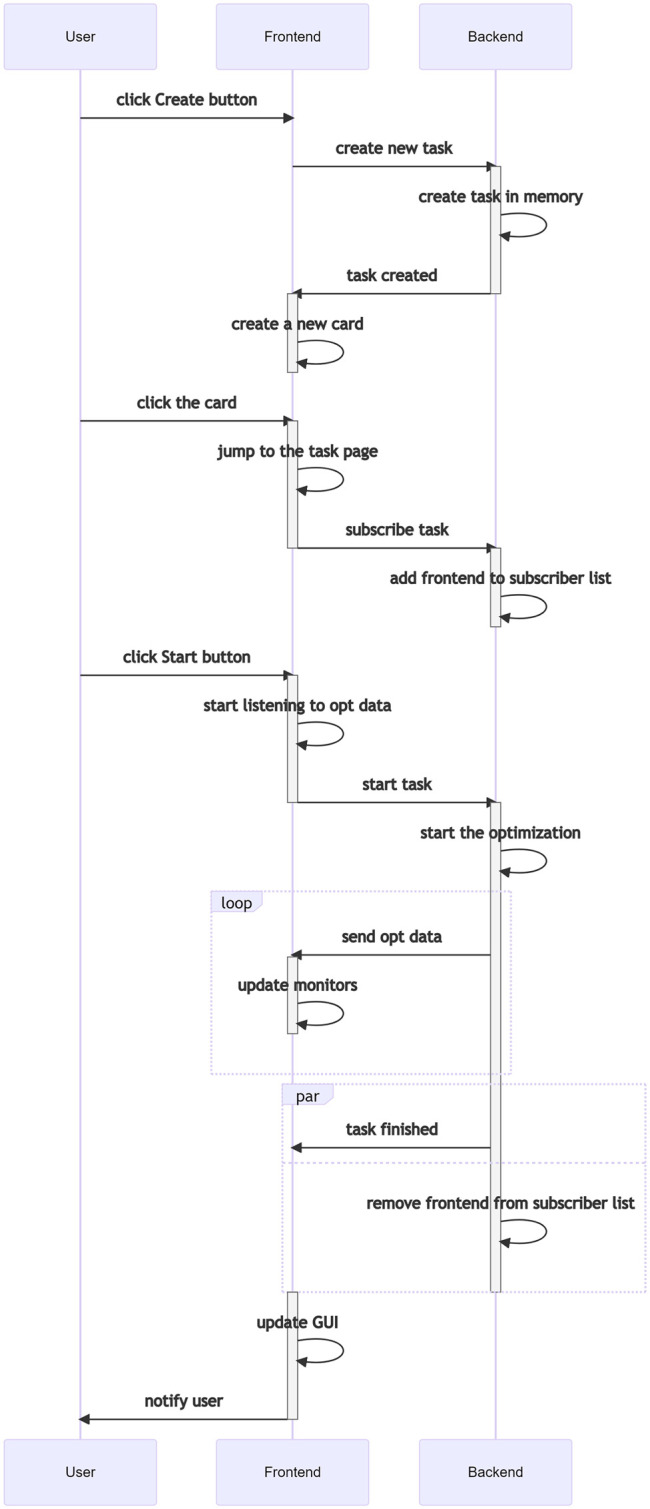
Signal sequence when a user creates and runs an optimization task through the built-in Teeport GUI.

The default Teeport frontend connects to the Teeport backend service and sends and receives messages over the socket. Take the following scenario as an example. A user wants to create a new task with optimizer A and evaluator B, then monitor the optimization process. To do this, the user clicks the “New Task” button and selects/configures optimizer A and evaluator B. The user then clicks the “Create” button, which sends a Create a new task message to the backend. The backend responds with a Task created message when the task initialization is done. Once the frontend receives the Task created message, it should create a new card on the GUI to notify the user that the task has been initialized. When the user clicks the new card, a Subscribe the task message will be sent to the backend and the frontend will be able to receive all the updates of the task from now on. When the “Start” button is clicked, a Start the task message would be sent and the optimization begins. The Teeport frontend gets all the optimization data generation by generation and visualizes the data on the fly. The user sees the optimization process in real-time. The process described above is visualized in Figure 9.

This fully-decoupled design of Teeport enables flexible interaction between the user and the optimization task. The actions that the user could do are not limited by the built-in GUI–instead, they can make use of the APIs provided by the Teeport backend service to achieve the goals, for example, exporting the optimization data and performing their own data analysis and visualization.

### 3.4 Flexible Deployment

Teeport was designed with a range of typical use cases in mind. When the users are dealing with some local optimization problems, Teeport should work as a normal local package that provides the tools that help the users to connect their optimizers and evaluators, without the burden to set up a server, configure domain names, etc. When a cross-lab optimization is needed, say, optimizing some property of a machine in one laboratory with an algorithm developed and deployed in another laboratory, Teeport should provide the options to be configured as a mature cloud-based service. Teeport is highly configurable regarding deployment. It could be deployed as simple as a local service that runs on the laptop, or it could be configured on the cloud to provide service across the internet. This flexibility in the deployment method enables a wide range of usage of Teeport under different circumstances.

## 4 Features

Teeport is an optimization platform, and thus provides the features that are commonly available in an optimization platform: integrating new algorithms/test problems, running optimizations with particular configurations, logging/viewing the optimization data, etc. However, Teeport has several special properties that distinguish it from the rest. A few signature features of Teeport would be discussed in the following paragraphs.

### 4.1 Effortless Integration

A big difference between Teeport and other optimization platforms is that Teeport doesn’t ship with any built-in evaluators and optimizers. Instead, Teeport provides excellent integrationality. As discussed in Section 2.6, Teeport only requires the users to wrap their algorithm/problem into a function with a specific signature. The users don’t have to learn the way that how the platform works to integrate their optimization code. Instead, they only need to focus on the core logic of their algorithm/problem, and shape the code to a better and more natural form (the optimize, evaluate, and process functions). For most cases we have faced, it only takes a few minutes to reorganize the code into a function that Teeport requires, so the user could get their optimizer and evaluator running on Teeport and perform the optimization with minimal effort. This effortless integration nature of Teeport enables itself to function as a bridge that connects different kinds of algorithms and problems since it’s much easier to integrate both sides into Teeport, rather than adopt one side to the other. Thanks to the effortless integration feature, a large number of optimization algorithms and test problems from various platforms, such as PyGMO (Biscani and Izzo, 2020), pymoo (Blank and Deb, 2020), PlatEMO (Tian et al., 2017) and Ocelot Optimizer (Tomin et al., 2016), have already been integrated into our cloud-hosted Teeport instance.

### 4.2 Anonymous Optimizer/Evaluator

For a typical optimization platform, the algorithm/problem code has to be put into the code base of that platform before being used. This could bring some troubles. Sometimes the evaluation code contains confidential information, such as the token to communicate with the machine control system or the critical internal control knobs that could be dangerous to expose. Teeport solves this issue by making the optimizer/evaluator anonymous. The implementation details of the algorithm and the problem remains unknown to Teeport during the whole life cycle of an optimization process. The optimize/evaluate function that is passed to the Teeport APIs just provides Teeport a way to call the optimizer to do the optimization/evaluator to do the evaluation. The actual computation/measurement happens where the optimizer/evaluator lives, not on the server that hosts the Teeport backend service. This anonymity of the optimizer/evaluator guarantees that the implementation details of the algorithm/problem would not be unnecessarily exposed, as well as prevents unintentionally changing to the evaluation code which may lead to serious consequences.

The way that Teeport integrates the optimizer/evaluator also grants users full ownership and control over the shipped optimizer/evaluator. Imagine a remote collaboration scenario where user A applies an algorithm to deal with a machine property optimization problem provided by user B through Teeport. Then user B decides when to start/stop running the evaluator, user A has no control over the evaluator shipped by user B, which makes it impossible for the optimizer to accidentally changing the machines operation through the evaluator.

### 4.3 Embeddability

Teeport is not only designed to be a platform on which the users integrate their optimizers and evaluators then perform optimizations, but also a flexible solution that could be inserted into the user’s existing workflow without much effort and interruption. It is normal that the user already built an optimization workflow while struggling to add new algorithms to the workflow, due to various limitations. Teeport could help in this case by acting as a proxy–just integrate the algorithms of interest into Teeport first, then use the use_optimizer (id: string) API to select the corresponding algorithm, and use the Teeportized optimize function in the workflow. Teeport would not force the users to run everything on itself, instead, Teeport tries to help the users to solve their optimization communication/integration problems–by properly extending the existing system. Embeddability also means that Teeport supports nested optimizers/evaluators. Specifically, one could do hyper-parameters tuning with a nested optimizer/evaluator setup. Let’s denote optimizer A as the optimizer of which the hyper-parameters to be tuned, evaluator B as the test evaluator on which to run optimizer A with a specific set of hyper-parameters, and optimizer C as the optimizer used to tune the hyper-parameters of optimizer A. Then we could make an evaluator D of which the input being the hyper-parameters of optimizer A, once evaluating, an optimization task with optimizer A and evaluator B will be created and run, then several performance indicators would be calculated and returned as the output. Finally, creating and running an optimization task with optimizer C and evaluator D would get the hyper-parameters tuning task done. One caveat of this approach is that each time a new set of hyper-parameters is proposed by optimizer C and evaluated on evaluator D, a new optimization task with optimizer A and evaluator B would be created and run. Therefore if 100 points were evaluated by evaluator D, there would be 100 sub-tasks created and run, along with the main task.

### 4.4 Auto-Granted Visualization and Control

When doing optimization with Teeport, the optimization data flow through the Teeport backend service. The Teeport backend service provides a set of APIs to hold/release the data flow and forward the data flow. The built-in Teeport GUI makes use of these APIs to grant visualization and control abilities to the users. Once the users ship the optimizer/evaluator by the Teeport adapter and start an optimization, they will be automatically granted a set of features through the Teeport GUI, such as monitoring the optimization progress, pausing, or resuming the optimization, terminating the optimization, and so on. Figure 2 left plot shows a monitored single objective optimization task, the user could use the toolbar to control the optimization process.

### 4.5 Easy-Comparison Among Multiple Runs

Teeport stores the recent optimization tasks in memory and archives the older runs. So the data of an optimization task that has been performed through Teeport would never get lost. Teeport supports lazy-loading of the history data, which means Teeport would load the optimization data from the database when it’s needed. The built-in Teeport GUI provides a feature that lets users select multiple optimization tasks (including the actively running ones) and compare the multiple runs in the same frame. With this capability, users can easily compare the performance of an optimizer on a series of testing evaluators, or compare the efficiency of different optimizers against the same to-be-optimized evaluator, as shown in the right plot in Figure 2.

Through the comparison feature of Teeport, the user could determine how efficient each optimizer could be for the specific evaluator on the fly during the experiment, and adjust our optimization strategy accordingly.

### 4.6 Background Algorithm Benchmarking

Once a local optimize function is shipped as an optimizer to Teeport, Teeport knows how to run it, stop it, and handle exceptions. Based on these basic control, Teeport provides the functionality to run the optimizer multiple times against a test evaluator and collect the data of the multiple runs to analyze the algorithm performance. Teeport backend supports to run an optimization task in the benchmarking mode, and the users could get several meaningful performance visualizations (such as objective mean and variation, Pareto front distribution for multi-objective optimizations, etc) through the built-in Teeport GUI. Users could also query the benchmark run data from the Teeport backend and perform customized data analysis. The multiple runs that are needed to do the benchmark are run in the background and managed by the Teeport backend service. The user only needs to initialize the benchmark task then forget about it. Once the benchmark is done, the user could come back and check out the visualized result or collect the data.

### 4.7 Optimization Tasks Management

A task in Teeport not only contains the optimizer/evaluator information and the history data, but also metadata such as task name, task description, and task creation/completion time. With these metadata embedded, the user could easily filter out the tasks of interest (such as all optimizations run with some specific optimizer within a particular date range) with the search query API of Teeport.

Teeport also provides the data exporting/importing functionalities so the users completely own their data and wouldn’t be locked within the Teeport framework. Teeport can be used as a pure optimization runner where the users extract the optimization data afterward and perform their own analysis/visualization; It could also be used as an optimization data analysis/visualization tool that takes the imported data and lets users explore/compare the optimization runs.

### 4.8 Planned Features: Breakpoint Recovery

If anything can go wrong, it will. Murphy’s law is particularly correct for a remote online optimization scenario. Teeport relies on the network to exchange information between the evaluator and the optimizer. What if the network connection was interrupted? What if the evaluator crashed in the middle of the optimization? What if the experimental condition went south and produced several invalid points? The usual answer would be doing the optimization again, however for the online optimization case, measurement on one point is usually expensive–either in terms of time or money, sometimes both. A much better way to deal with these bad situations is to roll back the optimization process to the latest checkpoint and continue from there. The architecture of Teeport makes breakpoint recovery and “time-traveling” realistic. By reconstructing the algorithm in a loop-basis way as shown below:

$$state=loop\left(evaluate,state,con\mathrm{fi}gs\right)$$ (4)

**FIGURE 10:**
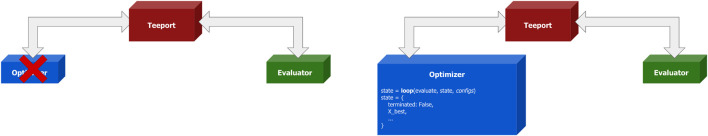
Breakpoint recovery in Teeport. Left: The optimizer crashes in the middle of optimization, which breaks the whole optimization process; Right: A loop-basis optimizer could recover the optimization from the breakpoint.

Where state is the internal state of the algorithm, evaluate and configs share the same definition as in Eq. 2, in each loop the algorithm performs calculations and updates its internal state, the loop is repeated until meeting the termination condition. Teeport caches the internal states during the optimization process loop by loop, and if anything bad happened, Teeport could simply backtrack the optimization to the last loop that everything worked, then resume the optimization from there. The breakpoint recovery idea is visualized in Figure 10.

The relationship between the regular form of an optimize function as shown in Eq. 2 and the loop-basis form as shown in Eq. 4 is explained in the pseudo code below.

**def** optimize (evaluate, configs):
state = configs [‘init_state’]. copy ()
**while not** state [‘terminate’]:
state = loop (evaluate, state, configs)

The optimize function is basically composed by a sequence of the loop function calls. The loop function determines the granularity of the optimization backtracking system–it is the smallest unit of that backtracking system. The reason why the optimizer has to be rewritten in the loop-basis form to support the backtracking feature is that Teeport has no access to the internal states of the optimizer once the optimize function is called, so it would not be possible to tell the optimizer to go back to a history state since Teeport does not have that information. A loop-basis form would resolve this issue by exposing the internal states of the optimizer to Teeport. Embedding the state information into the configs argument of the regular form optimize function would not work, due to the fact that only X and Y are forwarded each generation by Teeport, not the configs^3^, so Teeport is not aware of the changes in the configs.

Breakpoint recovery and time-traveling is a planned feature for Teeport, which would be implemented and tested soon.

## 5 Applications

### 5.1 Remote Online Optimization

One of the most straightforward applications of Teeport is doing online optimization remotely. In the accelerator field, the big machines that generate, accelerate, and store the electron/proton beams usually require a highly complicated control system to work. Most of the work that regarding interacting with the control system occurs in the accelerator control room (ACR). The routine to perform an online optimization task on these big machines is as follows: 1) Clone the algorithm from the local computer to the computer in the ACR; 2) Adapt the algorithm to work with the evaluation script of the property to be optimized; 3) Run the algorithm in the ACR and wait there until the optimization is done.

With Teeport, performing an online optimization remotely is effortless. Below is the routine of the online optimization of the SPEAR3 beam loss rate with Teeport (Zhang et al., 2020):

1. Run the beam loss rate evaluation script as an evaluator through the Teeport adapter for Matlab in the ACR
2. Get the corresponding local evaluator through the Teeport adapter for Matlab on the local laptop
3. Call the optimize function with the local evaluate function

More details can be found in the corresponding paper (Zhang et al., 2020).

In general, performing a remote online optimization is as simple as doing a local optimization with Teeport. The workflow to do an online optimization remotely usually looks as follows:

1. Code the experimental evaluator, and integrate it to Teeport with the run_evaluator API. Teeport will generate an Id and assign it to the evaluator.
2. On the local computer, use the Teeport adapter for the language of the optimizer, and get a local evaluate function through the use_evaluator (id) API, with the Id from the last step.
3. Call the local optimize function on the local evaluate function to perform the optimization.

**FIGURE 11:**
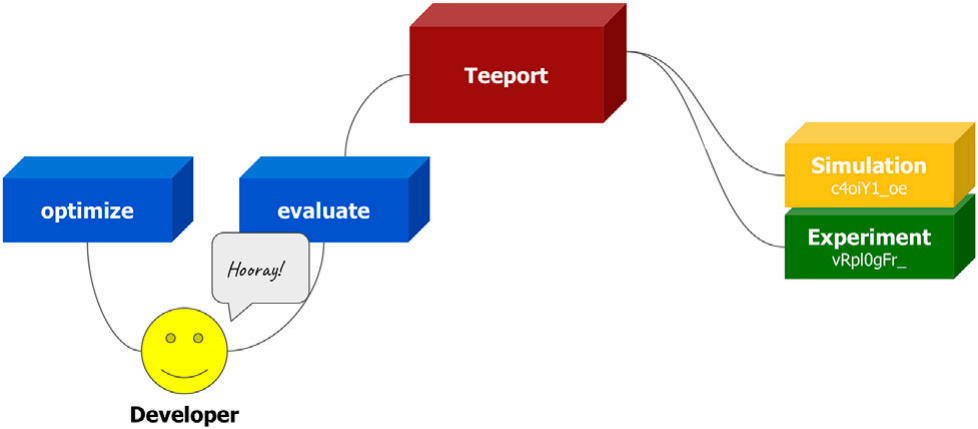
Fast switching between the simulation evaluator and the experimental evaluator. The user can select the evaluator to be optimized upon by the corresponding id, get the specific local evaluator through the use_evaluator (id) API, and perform the optimization.

After going through the above steps, the user will be automatically granted a set of nice features through the Teeport GUI, such as monitoring the optimization progress, pausing or resuming the optimization, terminating the optimization, and so on. Teeport also makes it easier for the user to do several dry runs before performing the real online optimization. The only action needed is to change the evaluator id in the use_evaluator (id) API accordingly. This process is visualized in Figure 11.

### 5.2 Use Packages in a Different Language During Algorithm Development

During algorithm development, it’s almost indispensable to use well-written packages to boost the development process. However, it’s also common that the best package for a specific task appears to be not available in the language of the algorithm. Since Teeport is capable to convert an arbitrary function to an online processor^4^, it could be applied in this case to “borrow” the functionalities of a package in a different language.

**FIGURE 12:**
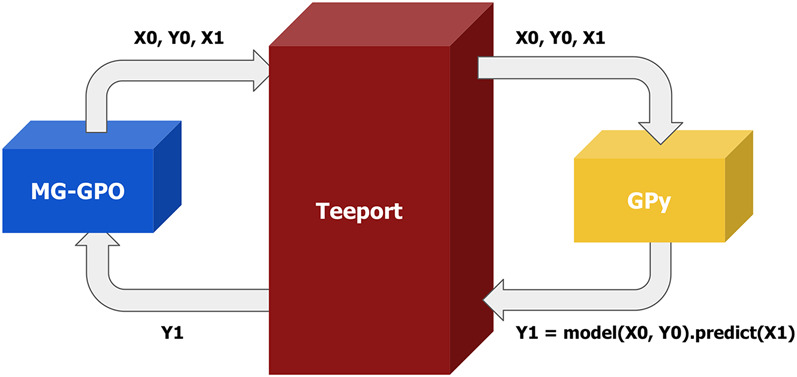
Use the functionality from the GPy Python package in the Matlab MG-GPO algorithm with Teeport. A process function is implemented in Python to make use of the modelling and predicting features provided by the GPy package. The needed data (**X0, Y0, X1**) to build the GP model is passed to the processor through the Matlab version of the process function from the use_processor (id) API and the prediction (**Y1**) is returned to the MG-GPO algorithm.

When we were developing MG-GPO (Huang et al., 2020), initially we were not able to find a good Matlab Gaussian process package, which is important as the GP modeling part is at the core of the algorithm. In Python, there does exist an excellent GP package called GPy (GPy, 2012, since). The problem was, how to use GPy to handle the GP modeling part while keeping all other logic in Matlab? This is solved with Teeport by running GPy’s GP modeling function as a processor on Teeport, applying the use_processor API to get a Matlab version of the GP modeling function, and using it in our algorithm evolution loop. This approach is demonstrated in Figure 12.

### 5.3 Extend the Ability of Other Optimization Platforms

Ocelot optimizer (Tomin et al., 2016) is a platform for automated optimization of accelerator performance. There are two core abstractions in Ocelot: the machine interface that communicates with an accelerator control system and contains all machine-related specifics, and the optimization method that does the optimization with a few built-in optimization algorithms. Ocelot optimizer works great in the ACR, while it has one drawback: it’s hard to integrate a new machine interface or an optimization method into the Ocelot optimizer since the integration process involves some non-trivial modifications to the Ocelot source code. Teeport was used as a plugin to tackle this issue.

**FIGURE 13:**
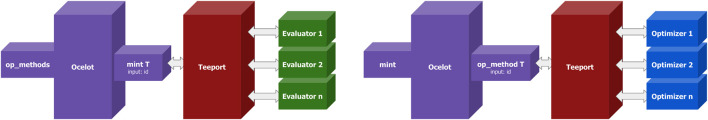
Teeport as an Ocelot plugin. Left: Extending the machine interfaces in Ocelot through Teeport; Right: Extending the optimization methods in Ocelot through Teeport.

The idea is shown in Figure 13. The Ocelot source code was modified once to integrate with the Teeport machine interface and the Teeport optimization method, then all the new machine interfaces (the evaluators) and the optimization methods (the optimizers) can be integrated into Teeport, and being called from the Ocelot optimizer through the use_evaluator (id) and use_optimizer (id) APIs.

There is an alternative approach that instead of using Teeport as an Ocelot plugin, the Ocelot optimizer is used as a plugin for Teeport. In this way, all the built-in optimization methods and machine interfaces can be used by the external evaluators and optimizers, accordingly, to perform the optimization task.

### 5.4 Work as a Standard Algorithm Benchmarking Platform

When developing new algorithms, the developer usually needs to benchmark the newly developed algorithm against a set of standard test problems implemented in the same language as the algorithm. The benchmarking results obtained in this way, unfortunately, could be vague, confusing, and misleading from time to time, therefore degrades the convincibility of the algorithm performance. This is caused by multiple slightly different implementations of a test problem, and it’s sometimes not clear how the parameters of the test problems were set when performing the benchmark. If there is a standard benchmarking platform that provides various test problems with clear definitions/descriptions of the internal parameters, then the benchmarking result would be more trustworthy, since it’s provided by a third-party test platform. The platform should also provide a simple way to let developers ship and test their algorithms without too much effort.

A cloud-hosted Teeport is a perfect candidate for this application. Optimizers and evaluators on Teeport are anonymous, so the developer would not need to worry about exposing the unpublished algorithm. The standard evaluators cannot possibly be modified outside of the server that hosts them (which should be kept in a secret place), so the benchmarking process with Teeport is strict and fair. When reporting the algorithm performance in the paper, the benchmarking result could be referred to by its task id. With the task id, one (most likely the reviewer of the paper) could locate the corresponding benchmark runs on Teeport and verify the algorithm performance easily. With Teeport as a standard algorithm benchmarking platform, it would be much easier for the developer to benchmark the newly developed algorithm, as well as for the user/reviewer to verify the stated performance of the algorithm of interest.

## 6 Conclusion

We developed a real-time communication-based online optimization platform, Teeport, to break the communication wall between the optimization algorithms and the application problems that live in different environments or written in different languages. Teeport abstracts various algorithms and problems as functions with specific signatures, which are called optimizers and evaluators, respectively. Teeport provides a set of APIs to let the users effortlessly ship their optimizers and evaluators. Once the optimizers and evaluators are shipped to Teeport, the users are automatically granted a rich feature set through the built-in Teeport GUI, including optimization process controlling, monitoring, and benchmarking. A large number of optimization algorithms and test problems from various platforms, such as PyGMO, pymoo, PlatEMO, and Ocelot Optimizer, have been integrated into Teeport. Teeport can be used as a local package or deployed as a cloud-hosted service to enable remotely optimization collaborations. We applied Teeport to perform and control remote online optimizations, monitor and benchmark the performance of the optimization algorithms, and help to develop and enhancing algorithms. Teeport has been tested and deployed at SLAC and ANL. We plan to implement the optimization rollback feature in Teeport soon.

## Data Availability

The source code of Teeport can be found in the following repositories: Teeport backend: https://github.com/SPEAR3-ML/teeport-backend, Teeport frontend: https://github.com/SPEAR3-ML/teeport-frontend, Teeport Python adapter: https://github.com/SPEAR3-ML/teeport-client-Python, Teeport Matlab adapter: https://github.com/SPEAR3-ML/teeport-client-matlab, Teeport plugins: https://github.com/SPEAR3-ML/teeport-plugins. More documentations of Teeport are available in the following links: Teeport introduction slides: https://teeport.ml/intro, Teeport tutorial: https://github.com/SPEAR3-ML/teeport-test, Teeport API docs: https://teeport-client-Python.readthedocs.io. A demo website is also available at: Teeport demo: https://teeport.ml/tasks.

## Footnotes

1 Usually happens when the algorithm that calls the evaluate function is not an evolutionary algorithm.

2 For the scenarios that require complex number arrays, the real part and imaginary part of the complex number array will be stored in two real number arrays and combined into a complex number array again when the data reach the target.

3 configs is only forwarded once at the beginning of the optimization task.

4 As long as the function is pure (Jones, 2003) and the arguments and returns of the function are serializable.
